# Absolute oral bioavailability, quantitative toxicokinetics and metabolite profiling of alternariol and alternariol monomethyl ether in pigs

**DOI:** 10.1007/s00204-025-04050-y

**Published:** 2025-04-26

**Authors:** Danica den Hollander, Siegrid De Baere, Celestien Holvoet, Mathias Devreese, Gunther Antonissen, Ann Martens, Kristel Demeyere, Kris Audenaert, Evelyne Meyer, Siska Croubels

**Affiliations:** 1 Laboratory of Pharmacology and Toxicology, Department of Pathobiology, Pharmacology and Zoological Medicine, Faculty of Veterinary Medicine, Ghent University, Salisburylaan 133, 9820 Merelbeke, Belgium; 2 Laboratory of Biochemistry, Department of Veterinary and Biosciences, Faculty of Veterinary Medicine, Ghent University, Salisburylaan 133, 9820 Merelbeke, Belgium; 3 Chair Poultry Health Sciences, Department of Pathobiology, Pharmacology and Zoological Medicine, Faculty of Veterinary Medicine, Ghent University, Salisburylaan 133, 9820 Merelbeke, Belgium; 4 Department of Large Animal Surgery, Anaesthesia and Orthopaedics, Faculty of Veterinary Medicine, Ghent University, Salisburylaan 133, 9820 Merelbeke, Belgium; 5 Laboratory of Applied Mycology and Phenomics, Department of Plants and Crops, Faculty of Bioscience Engineering, Ghent University, Valentin Vaerwyckweg 1, 9000 Ghent, Belgium

**Keywords:** ADME, Alternariol, Alternariol monomethyl ether, Biomarkers of exposure, In vivo, Mycotoxins, Pig model, Risk assessment, Toxicokinetics

## Abstract

The mycotoxins alternariol (AOH) and alternariol monomethyl ether (AME), produced by *Alternaria* spp*.*, are common contaminants of food and feed and are a potential threat to animal and human health. To date, the most prominent data gaps for their comprehensive risk assessment concern information on their in vivo absorption, distribution, metabolism and excretion (ADME) and toxicokinetic behavior. The aim of this study was to determine the absolute oral bioavailability, quantitative toxicokinetic characteristics and biotransformation of AOH and AME in vivo in pigs, using crossover trials with intravenous and oral administration of a single dose of both mycotoxins at 2 mg/kg b.w. Plasma profiles of the mycotoxins and phase I and II metabolites were studied in the *vena jugularis* using UPLC-MS/MS and LC-HRMS methods. Furthermore, plasma from the *vena portae* was analyzed to study presystemic biotransformation. Urine was collected to determine the urinary excretion and metabolite profiles. Results reveal a low absolute oral bioavailability of AOH (15%) and AME (9%), caused by a low absorption and/or extensive first-pass biotransformation in the liver to mainly phase II, and to a lesser extent phase I metabolites. Quantitative toxicokinetic modeling of the IV data showed a high total body clearance for both AOH and AME (12.9 and 16.8 L/(h*kg b.w.), respectively), a high volume of distribution (4.97 and 5.15 L/kg b.w., respectively) and a short elimination half-life of 0.16 and 0.21 h, respectively. These findings may contribute to the risk assessment of AOH and AME and to the development of candidate biomarkers of exposure in biomonitoring studies since pigs are considered a suitable animal model to extrapolate to humans.

**Supplementary Information:**

The online version contains supplementary material available at 10.1007/s00204-025-04050-y.

## Introduction

Fungi of the genus *Alternaria* are well-known plant pathogens, and are worldwide causing considerable crop yield losses (Saleh et al. 2024). Some of their toxic secondary metabolites or mycotoxins are important food contaminants, mainly found in cereals, oilseeds, fruits and vegetables, posing a potential threat to both animal and human health. Comprehensive reviews indicate that occurrence data for *Alternaria* mycotoxins, as well as their hazard identification and characterization are mostly available for alternariol (AOH), alternariol monomethyl ether (AME) and tenuazonic acid (TeA) (Fraeyman et al. 2017; Mihalache et al. 2023; Louro et al. 2024; Saleh et al. 2024). A whole array of toxic effects has been uncovered for some of these mycotoxins, showing cytotoxic, genotoxic, mutagenic, immunosuppressive and endocrine disruptive properties (Louro et al. 2024). Despite these data, the European Food Safety Authority (EFSA) could not yet perform a risk assessment to set risk-based guidance values in food and feed, because of important data gaps. Hence, these mycotoxins are still considered as emerging toxins. Aichinger et al. (2021) concluded that the data gaps are not attributed to a lack of toxicity or occurrence, but to the complexity of these compounds and their diversity concerning chemical stability, bioactivities; absorption, distribution, metabolism and excretion (ADME) properties and exposure levels (Aichinger et al. 2021).

The risks for animal and human health as well as the dietary exposure assessment in the European population were addressed by EFSA (EFSA Panel on Contaminants in the Food Chain 2011; EFSA et al. 2016). Concerning animals, it was concluded already in 2011 that the knowledge of toxic effects in farm and companion animals and occurrence data in animal feed were insufficient to assess the health risk for different animal species (EFSA Panel on Contaminants in the Food Chain 2011). More recently, the worldwide frequent occurrence of AOH, AME and TeA in finished pig feed samples (n = 524) was demonstrated, showing respectively 70%, 59% and 73% of the samples were contaminated (Khoshal et al. 2019). Furthermore, the in vivo exposure of pigs was demonstrated by the analysis of urine samples (*n* = 56) collected from pigs before slaughter, showing a prevalence of AOH and AME in 54% and 73%, respectively (Tkaczyk and Jedziniak 2021). Nonetheless, only a few studies focus on the toxicity in pigs, and these did so only based on in vitro experiments. It was shown that AOH affects gene expression on a translational level in porcine endometrial cells (Wollenhaupt et al. 2008). It also disturbs porcine oocyte maturation and preimplantation development (Schoevers et al. 2020), while both AOH and AME negatively affect progesterone synthesis in porcine granulosa cells (Tiemann et al. 2009). Regarding porcine intestinal health, AME induces cell death and oxidative stress in swine intestinal epithelial cells (Marin et al. 2024).

With respect to human health, EFSA estimated that the highest chronic exposure of AOH and AME was present in toddlers (means of 3.8–71.6 ng/kg body weight (b.w.) per day for AOH and 3.4–38.8 ng/kg b.w. per day for AME), with the highest contribution from fruit and fruit products for AOH and vegetable oil and pears for AME (EFSA et al. 2016). The highest exposure to TeA was also estimated in toddlers with a mean exposure between 100 and 1614 ng/kg b.w. per day, with cereal-based food for infants and young children as the main contributor. These exposure values exceed the Threshold of Toxicological Concern (TTC), which is set at 2.5 ng/kg b.w. per day for AOH and AME based on their potentially mutagenic and/or carcinogenic properties, while for TeA having no evidence of genotoxic potential, the TTC has been set at 1500 ng/kg b.w. per day. Similar estimated dietary exposure exceeding the corresponding TTC values for AOH, AME and TeA was reported for Chinese infants and young children, with cereals and cereal-based infant foods as the primary contributors to their mycotoxin exposure (Ji et al. 2024). The European Commission (EC) therefore recommends further studies and controls on foodstuffs, particularly those intended for consumption by children. Hence, the EC issued indicative levels for AOH, AME and TeA above which investigations should be performed on the factors leading to the presence of *Alternaria* toxins or on the effects of food processing (EC 2022). In particular, the presence of processed tomato products, paprika powder, sesame seeds, sunflower seeds and oil, tree nuts, dried figs and cereal-based foods for infants and young children should be monitored. This recommendation further supports the need for additional compound-specific toxicity data as a prerequisite for a comprehensive risk assessment.

Louro et al. (2024) identified major data gaps to improve the risk assessment for human health and concluded important information on ADME processes is still missing. To date, mainly in vitro studies explored the phase I and II metabolism of selected *Alternaria* toxins using hepatic and intestinal microsomes from several species (rat, pig, human), as well as rat liver slices and cytosol fractions, and using differentiated Caco-2 cells (Burkhardt et al. 2009, 2011; Pfeiffer et al. 2008, 2009). However, only a limited number of in vivo studies is available characterizing ADME processes, using mainly mice and rats. These showed a low oral bioavailability for AOH and AME with extensive first-pass effect (Schuchardt et al. 2014; Puntscher et al. 2019). More specifically, Louro et al. (2024) assumed a high hepatic first pass since the intestinal absorption was rather high based on the apparent permeability in Caco-2 cells. On the other hand, the authors also concluded that gut microbiota may contribute to a reduced fraction available for absorption, based on earlier reported decreased recoveries in incubation experiments with several human gut bacterial strains (Crudo et al. 2020). Hence, metabolite profiling in both blood from the *vena portae* and systemic blood could bring more conclusive results about the site of presystemic mycotoxin metabolism. Concerning tissue distribution, no data on the volume of distribution (V_d_) have been reported, whereas Fliszár-Nyúl et al. (2019) demonstrated that AOH binds with a higher affinity to rat serum albumin compared to human, bovine and porcine serum albumins. Since quantitative data on the primary toxicokinetic parameters V_d_ and total body clearance (CL) are lacking, an estimation of the elimination half-life (t_1/2el_) can currently only be done based on oral data. Hence, a study with intravenous (IV) administration could shed light on these toxicokinetic parameters, as well as on the absolute oral bioavailability. Such a study has been done by our group with TeA in male pigs and broiler chickens of mixed sexes, showing a complete absolute oral bioavailability in both species. In addition, marked species differences were seen, with a CL of 448.4 mL/(h*kg b.w.) and 59.3 mL/(h*kg b.w.), and a V_d_ of 325.8 mL/kg b.w. and 164.6 mL/kg b.w. in pigs and chickens, respectively, resulting in a t_1/2el_ of 0.51 h and 2.03 h, respectively (Fraeyman et al. 2015).

To the authors’ knowledge, no in vivo toxicokinetic data of AOH and AME are available in target animal species such as pigs. Pigs can also be considered a superior biomedical animal model compared to rodents because of their good resemblance with humans, both anatomically, physiologically and biochemically (Gasthuys et al. 2016). Likewise, piglets can be considered a promising potential animal model for children, based on the ontogeny and maturation of major organ systems involved in ADME processes (Gasthuys et al. 2017a; Millecam et al. 2018). Therefore the aim of this study was to determine the absolute oral bioavailability, full toxicokinetic characteristics and biotransformation of AOH and AME in vivo in piglets, using crossover trials with IV and oral administration.

## Materials and methods

### Mycotoxin standards and reagents

**Fig. 1:**
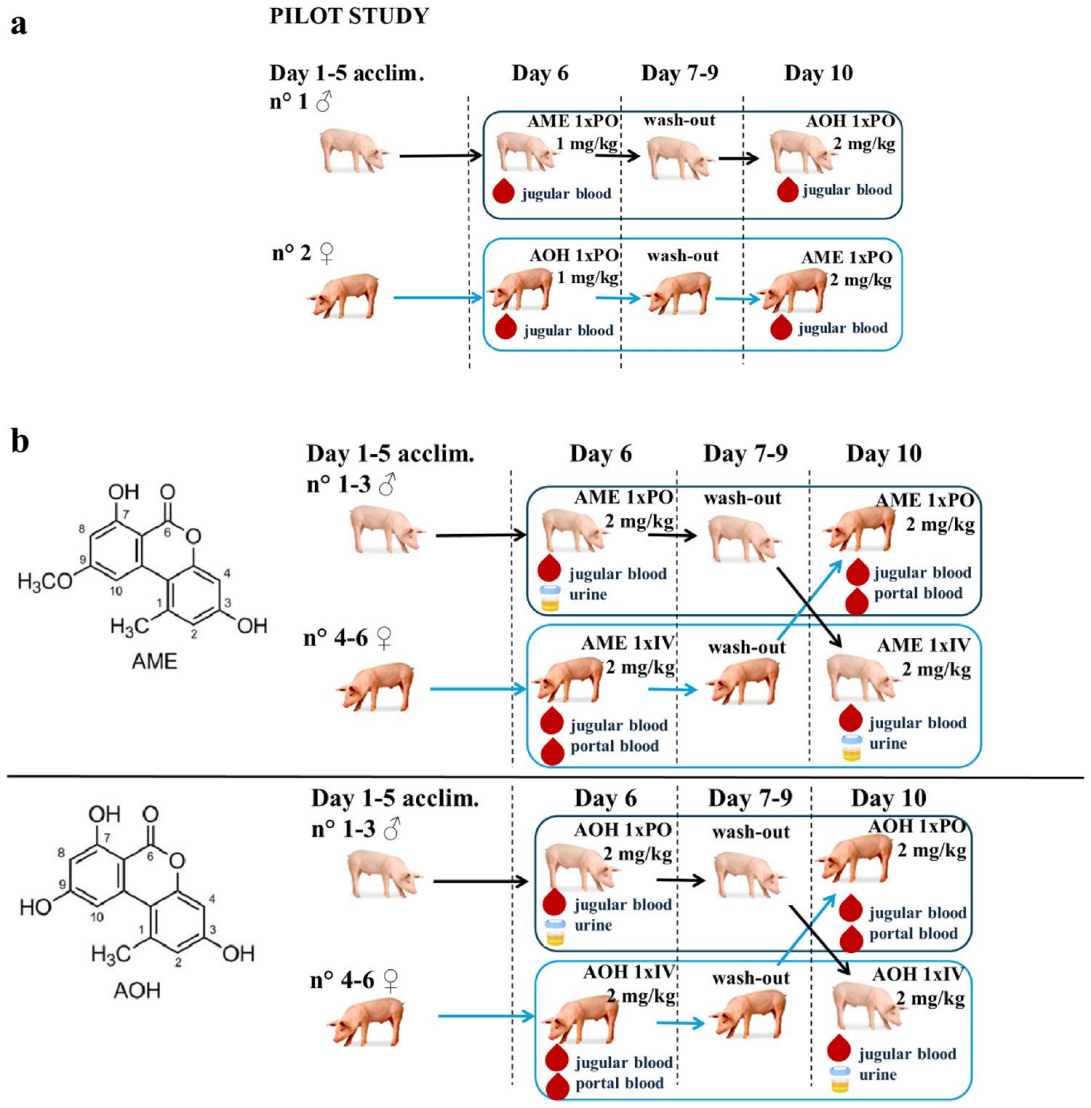
**a** Graphical representation of the study design of the pilot trial. **b** shows a graphical representation of the design of the final trials and the chemical structure of alternariol (AOH, bottom) and alternariol monomethyl ether (AME, top)

AOH and AME (Fig. 1) were purchased from Fermentek (Jerusalem, Israel). The internal standards (IS), alternariol-d4 [^2^H_4_] (AOH-d4) and alternariol monomethyl ether-d4 [^2^H_4_] (AME-d4) were a kind gift from Dr. Rychlik (Technical University of Munich, Germany; Asam et al. 2009). AOH, AME and IS were stored at ≤ − 15 °C.

Methanol (MeOH), acetonitrile (ACN), formic acid (FA), acetic acid (AA) and ammonium formate (NH_4_FA) were of LC–MS grade and obtained from Biosolve (Valkenswaard, The Netherlands). Dimethyl sulfoxide (DMSO), ethyl acetate (EtAc) and hydrochloric acid 37% (HCl) were of analytical grade and purchased from Sigma-Aldrich (Bornem, Belgium). Ultra-pure water was from a Milli-Q system (Merck-Millipore, Overijse, Belgium).

### Preparation of stock- and working solutions

For the administration of AOH and AME to pigs, stock solutions of AOH and AME (5 mg/mL) were prepared in DMSO. For the ultra-performance liquid chromatography − tandem mass spectrometry (UPLC-MS/MS) analysis of plasma and urine, stock solutions of AOH and AME (5 mg/mL) and their IS, AOH-d4 (50 μg/mL) and AME-d4 (25 μg/mL), were prepared in MeOH. Working solutions of 10, 1, 0.1, 0.01, and 0.001 μg/mL AOH or AME were prepared freshly for each experiment by appropriate dilution of the stock and working solutions with MeOH. A working solution (1 or 0.1 μg/mL) of IS was made by further dilution of the stock solution in MeOH. All stock- and working solutions were stored at ≤ −15 °C.

### Animal experiments

The animal trials were approved by the Ethical Committee of the Faculty of Veterinary Medicine and the Faculty of Bioscience Engineering of Ghent University (EC case number 2021/071). Care and use of the animals were in compliance with national legislation (Royal Decree 2013) and European Directive 2010/63/EU (European Union 2010). The whole study consisted of a pilot trial with 2 clinically healthy 4-week-old piglets to determine the mycotoxins dose, and two final animal trials for AME and AOH, each performed with 8 clinically healthy 4-week-old piglets of equally divided sexes (Seghers Hybrid^®^, RA-SE Genetics, Lokeren, Belgium). A graphical representation of the pilot trial and final trials is shown in Fig. 1a and b, respectively, including the number and sex of the piglets involved, the mycotoxins dose and routes of administration, and the types of samples collected. All piglets underwent an acclimatization period of 5 days in which they were housed together in a suitable pen, provided with straw, feed and water ad libitum. For all trials, commercial pig feed was analyzed for the presence of mycotoxins by a multi-mycotoxin LC–MS/MS method (Primoris, Zwijnaarde, Belgium). No AOH or AME were detected (limit of detection (LOD) of 2.0 µg/kg), but the feed for the AME trial contained 80.8 µg/kg deoxynivalenol (DON), whereas the feed for the AOH trial was contaminated with 139.0 µg/kg DON and 18.3 µg/kg zearalenone (ZEN). These levels were however below the maximum guidance levels of 900 µg/kg DON and 100 µg/kg ZEN (feed for piglets), as set by the EC (2006). Temperature of the enclosure was kept between 22 and 24 °C and natural light was present.

The pilot study for dose determination was performed with oral (PO) dosing of 2 piglets (Fig. 1a). One male piglet received a single AME dose of 1 mg/kg b.w. and 3 days later a single dose of AOH of 2 mg/kg b.w.; whereas another female piglet first received AOH at 1 mg/kg b.w. and 3 days later AME at 2 mg/kg b.w. A dose of 2 mg/kg b.w. was selected since this allowed constructing plasma concentration–time profiles with a sufficient high number of samples having levels of the parent mycotoxins above the limit of quantification (LOQ) after PO administration (unpublished data).

In both final trials, 6 out of 8 piglets were participating in the study, while the other 2 served as spare piglets. The mean (± standard deviation or SD) b.w. of all 16 piglets at arrival was 7.3 ± 1.4 kg. In all 6 piglets of each trial, a double-lumen central venous catheter was surgically introduced in the *vena jugularis* to facilitate repetitive blood sampling to construct rich-data plasma concentration–time profiles, as described by Gasthuys et al. (2009). Simultaneously, 3 (females) of the 6 piglets were provided with a single lumen *vena portae* catheter to study possible presystemic biotransformation of the mycotoxins. This portal vein surgery was performed by splenic catheterization via celiotomy, also as described by Gasthuys et al. (2009). The jugular vein plasma represents systemic plasma concentrations, i.e. after PO dosing taking into account a possible first-pass effect in both gut and liver. Whereas portal vein plasma results give information about a possible first-pass effect taking place in the gut only. The surgical procedures took place 2 days before mycotoxin administration, and all piglets received during these 2 days a daily dose of ceftiofur as preventive antibiotic therapy (Excenel flow®, Zoetis, Belgium) and meloxicam as anti-inflammatory drug (Metacam®, Boehringer-Ingelheim, Germany). After surgery, all piglets were housed individually for the prevention of reciprocal mutilation and removal of the catheters.

In the first trial, all 6 piglets (3 females, 3 males) were administered a single dose of AME at 2 mg/kg b.w. via PO and IV route, in a two-way crossover design. In the second trial, AOH was administered to 6 other piglets (3 females, 3 males) as a single dose of 2 mg/kg b.w. PO and IV, also in a two-way crossover design (Fig. 1b). For each trial, the 3 female piglets first received the IV dose via the smallest lumen of the double-lumen central vein catheter, while the 3 male piglets received the PO dose by gastric gavage using a stomach tube. After a wash-out period of 72 h, identical crossover administration was set up in the same animals, with the females and males then receiving the single PO and IV dose, respectively. The animals were weighed the day before administration, and were deprived from feed 12 h prior to administration until 4 h post administration (p.a.). The calculated volume of stock solution (5 mg/mL) for each animal was slowly injected in the central venous catheter for IV administration (followed by flushing with saline) and was diluted with saline for PO gastric gavage. After PO dosing, the gavage tube was flushed with 50 mL of tap water to ensure the full dose was administered. Jugular blood (using the largest lumen of the catheter) from all 6 piglets and portal blood samples from the 3 female piglets (1–2 mL/sample) were taken in EDTA tubes at following time points after IV and PO dosing: 0, 5, 15, 30, 60, 90, 120 min, 3, 4, 6, 8, 12, 24, 36, and 48 h p.a. Blood samples were kept refrigerated and were centrifuged (3000 rpm, 10 min, 4 °C) within 2 h after sampling. Plasma was then stored at −80 °C until further analysis.

For both trials, besides plasma also total volume urine samples were taken from the male piglets (*n* = 3) at the following collection intervals: 0–4, 4–8, 8–12, 12–24, 24–36 and 36–48 h p.a. after both IV and PO administration. A non-invasive urine sampling technique was used, based on the application of urine pouches (Gasthuys et al. 2017b). Urinary volumes at each collection interval were determined to construct the cumulative urinary excretion profiles, and samples were stored at −20 °C until further analysis.

### Quantitative UPLC-MS/MS analysis of AOH and AME in plasma and urine

#### Sample extraction

To 250 μL of pig plasma, 20 μL of a mix of IS working solution was added (WS_IS_mix_ 100 ng/mL), followed by vortex mixing and equilibrating for 5 min at room temperature. A volume of 750 μL of 0.1% FA in ACN was added, followed by vortex mixing (1 min) and centrifugation (10 min, 13,000 rpm). The supernatant was transferred to a 96-well sample collection plate (2-mL square well, Waters) and evaporated under a gentle nitrogen stream (~ 45 °C). The dry residue was reconstituted in 250 μL of water/MeOH (15/85, v/v), followed by vortex mixing for 15 s (2500 rpm). After covering the 96-well collector plate with a mat cap, a 1.0-μL aliquot was injected into the UPLC-MS/MS instrument.

To 500 μL of pig urine, 25 μL of a mix of IS working solution was added (WS_IS_mix_ 1 μg/mL), followed by vortex mixing and equilibrating for 5 min at room temperature. The urinary pH was checked using a pH paper (pH 0–14, Merck, Novolab, Geraardsbergen, Belgium) and adjusted to pH 2 using a 1 M HCl solution. Three mL of EtAc were added to the sample, followed by vortex mixing (1 min) and extraction for 15 min by rolling on a horizontal rotary apparatus. The sample was centrifuged (10 min, 4000 rpm) and the supernatant was transferred to another tube and evaporated under a gentle nitrogen stream (~ 45 °C). The dry residue was reconstituted in 250 μL of water/MeOH (15/85, v/v), followed by vortex mixing for 15 s (2500 rpm). After transferring the reconstituted extract to an autosampler vial, a 5.0-μL aliquot was injected into the UPLC-MS/MS instrument.

### UPLC-MS/MS analysis

Two different UPLC-MS/MS instruments were used for the quantitative analysis of AOH and AME in plasma and urine samples, a Xevo TQ-XS^®^ MS/MS and Quattro Ultima^®^ MS/MS system, respectively. This was based on the higher detection sensitivity needed for plasma, considering lower levels in plasma compared to urine.

The UPLC system for plasma analysis consisted of an Acquity UPLC H-Class + Quaternary Solvent Manager and Flow-Through-Needle Sample Manager with temperature controlled tray and column oven from Waters (Antwerp, Belgium). Chromatographic separation was achieved on an Acquity UPLC Phenyl-Hexyl column (150 mm × 2.1 mm i.d., dp: 1.8 μm) in combination with an Acquity Phenyl-Hexyl 1.8 μm Vanguard pre-column, both from Waters. The mobile phase A consisted of 0.1% AA in water, while mobile phase B was MeOH. A gradient elution was performed: 0–0.25 min (60% A, 40% B), 11.0 min (linear gradient to 90% B), 11.0–14.5 min (10% A, 90% B), 14.8 min (linear gradient to 60% A), 14.8–18.0 min (60% A, 40% B). The flow rate was 0.3 mL/min. It should be mentioned that the composition of the reconstitution solvent (water/MeOH (15/85, v/v)) not fully matched with the starting conditions of the chromatographic method (40% MeOH). Ideally, more similar conditions are used. Our sample preparation methodology was based on the protocol of a multi-mycotoxins LC–MS/MS method as described by Lauwers et al. (2019). According to this protocol, a reconstitution solvent of water/methanol (15/85, v/v) was the best compromise for the analysis of multiple mycotoxins with different physico-chemical properties. Nevertheless, as can be seen from the chromatograms in Fig. 5, this discrepancy between reconstitution solvent composition and initial chromatographic conditions did not result in peak distortion during chromatography. The temperatures of the column oven and autosampler tray were set at 40 °C and 8 °C, respectively. The UPLC column effluent was interfaced to a Xevo TQ-XS^®^ MS/MS system, equipped with an electrospray ionization (ESI) probe operating in the negative ion mode (all from Waters). A divert valve was used and the UPLC effluent was directed to the mass spectrometer from 3.5 to 14.5 min. Instrument parameters were optimised by direct infusion of working solutions of 100 ng/mL of all analytes and the IS at a flow rate of 10 μL/min and in combination with the mobile phase (50% A, 50% B, flow rate: 200 μL/min). The following parameters were used: capillary voltage: 2.7 kV, source offset: 30 V, source temperature: 130 °C, desolvation temperature: 550 °C, desolvation gas: 800 L/h, cone gas: 150 L/h, nebuliser pressure: 7.0 bar, LM resolution 1 and 2: 2.8 and 2.7 respectively, HM resolution 1 and 2: 14, ion energy 1 and 2: 0.4 and 0.8 respectively, collision gas flow: 0.15 mL/min. MS/MS acquisition was performed in the multiple reaction monitoring (MRM) mode. The MRM transitions for AOH, AME and their IS, as well as cone voltage, collision energy and retention times are shown in Table 1.

**Table 1:** MRM transitions and MS/MS parameters for alternariol (AOH), alternariol monomethyl ether (AME) and the internal standards (AOH-d4 and AME-d4)

Analyte	Precursor ion (*m/z*)^a^	Product ions (*m/z*)	CE^b^ (eV)	Cone (V)	Retention time (min)	Type of analysis^d^
AOH	256.95	212.90^c^ 184.90	22 25	25 25	7.05	Quan
AOH-d4	261.00	217.00^c^ 189.00	22 25	30 30	7.03	Quan
AOH-GlcA	433.10	175.00 257.00	23 23	25 25	5.95	Qual
AOH-Sulf	337.20	213.10 257.00	25 25	25 25	9.35	Qual
OH-AOH	273.00	214.00 258.00	23 23	25 25	5.50	Qual
AME	271.00	255.85^c^ 227.90	21 30	40 40	9.17	Quan
AME-d4	275.00	259.90^c^ 231.90	22 30	40 40	9.15	Quan
AME-GlcA	447.10	175.00 271.05	25 25	40 40	6.10 7.95	Qual
AME-Sulf	351.20	256.00 271.10	25 25	40 40	11.50	Qual

^a^*m/z* = mass to charge ratio; ^b^CE = collision energy; ^c^ion used for quantification; ^d^type of analysis: *quan* quantitative analysis, concentrations are reported; *qual* qualitative analysis, peak areas are reported

The UPLC system for urine analysis consisted of an Acquity Classic Binary Solvent Manager and Sample Manager with temperature controlled tray and column oven from Waters. Chromatographic conditions were the same as for UPLC-MS/MS analysis of plasma. The UPLC column effluent was interfaced to a Quattro Ultima^®^ MS/MS system, equipped with an ESI probe operating in the negative ion mode (all from Waters). A divert valve was used and the UPLC effluent was directed to the mass spectrometer from 3.5 to 14.5 min. The following parameters were used: capillary voltage: 2.7 kV, source offset: 30 V, source temperature: 130 °C, desolvation temperature: 400 °C, desolvation gas: 800 L/h, cone gas: 58 L/h, nebuliser pressure: 7.0 bar, LM resolution 1 and 2: 2.8 and 2.7 respectively, HM resolution 1 and 2: 15, ion energy 1: 1.0 and ion energy 2: 3.0, collision gas flow: 0.15 mL/min. MS/MS acquisition was performed in the MRM mode using the transitions as shown in Table 1.

### Method validation

Matrix-matched calibration curves and validation samples were prepared using an in-house stock of pooled plasma and urine samples from at least two control pigs receiving no AOH or AME. These control pigs were pigs from the same breed and of both sexes (Seghers Hybrid® from RA-SE Genetics (Lokeren, Belgium)). The age and size (b.w.) of the control pigs ranged from just weaned piglets (about 4 weeks of age, about 6 kg b.w.) up to about 8 weeks of age (about 20 kg b.w.). Plasma of both sexes was pooled to provide a stock of plasma. For urine, only urine from male pigs was pooled since urine was collected using non-invasive pouches which can technically only be placed on male pigs (Gasthuys et al. 2017b). Before using this pooled plasma and urine to prepare matrix-matched calibration curves and validation samples, a sample was tested to check the absence of the analytes studied.

The validation protocol and acceptance criteria were based on guidelines issued by the International Council for Harmonisation of Technical Requirements for Pharmaceuticals for Human Use (ICH 2022) and the International Cooperation on Harmonisation of Technical Requirements for Registration of Veterinary Medicinal Products (VICH 2015). Following validation parameters were evaluated: linearity, within- and between-run accuracy and precision, LOQ and LOD. The experiments for linearity and between-run accuracy and precision were performed over 3 days. The LOQ was determined using spiked samples, whereas the LOD was calculated using the results of the LOQ samples as the concentration that corresponds to a signal-to-noise ratio (S/N) of 3.

The results of the linearity (concentration range, correlation coefficient r and goodness-of-fit coefficient gof), within- and between-run accuracy and precision (the latter expressed as relative standard deviation or RSD), LOQ, and LOD for AOH and AME in plasma and urine can be found in Supplementary Table S1 and Table S2. The tables also show the acceptance criteria handled.

### Qualitative LC-HRMS analysis for metabolite profiling

Metabolite profiling was performed using multiple-stage and high-resolution mass spectrometry (HRMS). First, exact mass measurements of putative phase I and II metabolites in urine samples were carried out using LC-HRMS analysis. The urine extracts as prepared for the targeted UPLC-MS/MS analysis mentioned above, were injected on an Acquity I-Class UPLC instrument coupled to a Synapt^®^ G2-S*i* HDMS mass spectrometer (all from Waters). The chromatographic conditions were the same as described for the UPLC-MS/MS analysis. HR-MS instrument parameters were optimized by syringe infusion of a standard mixture solution of AOH and AME. The following HR-MS parameters were used: capillary voltage, 2.50 kV; sampling cone voltage, 40.00 V; source offset, 80.00 V; source temperature, 150 °C; desolvation temperature, 550 °C; cone gas flow, 150 L/h; desolvation gas flow, 800 L/h; nebuliser gas flow, 6.50 bar; lock spray capillary voltage, 2.50 kV. HR-MS acquisition was performed from 1.5 to 15.0 min in the negative ESI resolution mode using the MS continuum scan function. Time-of-flight (TOF) MS settings were as follows: low mass, 50 Da; high mass, 600 Da; scan time, 0.1 s; data format, continuum. The lock mass solution consisted of leucine encephalin (200 pg/μL). The lock spray was acquired during HR-MS acquisition, but no correction was applied. The lock spray settings were as follows: scan time, 0.215 s; interval, 30 s; scans to average, 3; mass window, 0.5 Da. Data processing and lock mass correction (*m/z* 554.262022) was performed using the Unify software (Waters). Identification of analytes was based on retention time (target T_R_ tolerance: 0.1 min) and mass (target mass tolerance: 10 ppm). The search for phase I and II metabolites of AOH and AME was performed using the ‘Accurate Mass Screening on MSe Data’ approach. The following transformations were added to the method (Supplementary Table S3), based on Burkhardt et al.^.^ (2011, 2012) and Pfeiffer et al. (2007): phase I transformations: reduction (+ H_2_), oxidation (+ OH), desaturation (-H_2_); phase II transformations: sulfation (+ SO_3_), glucuronidation (+ C_6_H_8_O_6_), methylation (+ CH_2_). Due to the lack of commercially available analytical standards for phase I and II metabolites, a qualitative approach was followed by presenting chromatographic peak areas only. Furthermore, Supplementary Table S3 shows acceptable mass error values of these compounds (both in mDa and ppm), calculated based on the theoretical and observed neutral mass.

For plasma metabolite profiling, the major metabolites found in urine were included in an additional qualitative analysis on the Xevo TQ-XS^®^ MS/MS instrument. Therefore, the monoisotopic mass of the precursor ion ([M-H]^−^) was calculated using the molecular weight calculator of the MassLynx software. Product ions were selected based on literature data (Appel et al. 2021; Burkhardt et al. 2011; Puntscher et al. 2019) and are shown in Table 1.

### Toxicokinetic modeling

The jugular plasma concentration–time data after IV administration were fitted to a one-compartmental (AME) or two-compartmental (AOH) toxicokinetic model with first-order elimination. Model fit was based on visual inspection of the goodness of fit plots, −2 log likelihood (−2LL) and precision of the parameter estimates. A model was only retained if it consistently demonstrated a robust fit across the different individual piglets. A multi-compartment model is generally more appropriate when the data suggest distinct distribution phases, whereas a one-compartment model suffices when the compound exhibits relatively uniform distribution and elimination behavior. It should be noted however that the number of compartments in a toxicokinetic model does not necessarily correspond to the compound’s actual physiological behavior, it is merely about fitting the available data. Hence, for a more mechanistic understanding, physiologically based pharmacokinetic (PBPK) modeling would be more appropriate, but this was outside the scope of the current study. The following toxicokinetic parameters were calculated based on IV administration: total body clearance (CL), volume of distribution at steady state (V_d_), plasma concentration at time 0 h (C_0_), elimination rate constant (k_e_) and elimination half-life (t_1/2el_). No model fitting was possible on the data derived from PO administration.

The absolute oral bioavailability (F, expressed as percentage) is defined as the percentage of the dose that is absorbed intact into the systemic circulation, and was calculated using the following equation:

$$F\left( \% \right) = \frac{{AUC_{0 - \infty PO } }}{{AUC_{0 - \infty IV } }} * 100$$

The areas under the plasma concentration–time curves from time 0 to infinity (AUC_0-∞_) were computed using the linear up-log-down trapezoidal method via non-compartmental analysis. Additionally, for PO data the descriptive toxicokinetic parameters maximal plasma concentration (C_max_) and time to C_max_ (t_max_) were given. For PO administration, data may deviate from the expected textbook profile due to substantial inter-individual variability in the absorption process. Given this variability, a non-compartmental analysis approach was chosen, as it allows direct analysis of the data without imposing structural model assumptions. This approach is not only equivalent but can be the preferred method in certain contexts, such as bioequivalence studies or studies comparing the toxicokinetic behavior of structurally related compounds. All data were processed using Phoenix 8.1 (Certara, Princeton, NJ, USA). Results are presented as mean values ± SD.

The urinary fraction excreted unchanged (f_e_) was calculated for each animal by dividing the cumulative amount of AOH or AME excreted in urine (A_r,∞_) by the dose of AME or AOH administered via IV route (2 mg/kg b.w.). The cumulative amount excreted (A_r,∞)_ was calculated by summing the amounts excreted during each individual collection interval (urinary conc * urinary volume).

## Results and discussion

### Plasma and urine toxicokinetics

Although a rather high dose of AOH and AME was administered, no adverse effects were noticed after either IV or PO single bolus dosing. Since both portal and jugular vein catheters were placed 2 days before mycotoxin administration, no influence of the surgical procedures was expected on the toxicokinetic behaviour of the compounds as the animals were fully recovered. Also, limited stress was observed when collecting blood via these catheters, in contrast to the stress following repeated direct venipuncture. As a technical remark, urine leakage from the collection bags was noted during the last sampling interval (36–48 h p.a.) for 2 piglets after IV AOH dosing, hence results of the amount excreted during this interval were based on one piglet only.

**Fig. 2:**
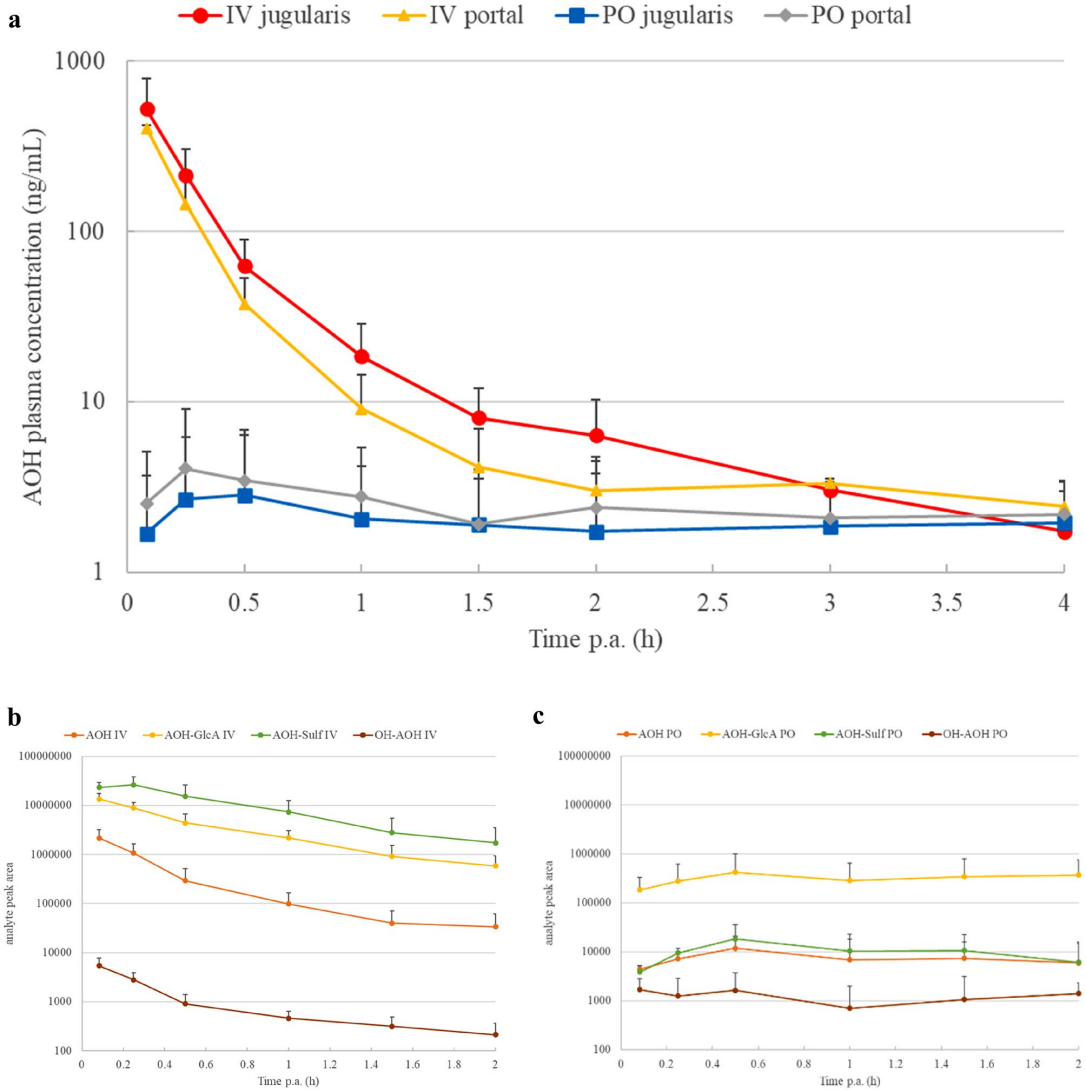
**a** Plasma concentration–time profiles (log-scaled) of alternariol (AOH) in piglets (*n* = 6) after a single intravenous (IV) and oral (PO) bolus dose administration of 2 mg/kg b.w. Blood was collected from both the *vena jugularis* (jugularis) and *vena portae* (portal). Values are presented as mean + standard deviation. **b** and **c** show the major phase I and phase II metabolite profile in jugular plasma after IV and PO dosing, respectively, and expressed as absolute chromatographic peak area (mean + standard deviation). OH-AOH: hydroxy-AOH; AOH-GlcA: AOH-glucuronide; AOH-Sulf: AOH-sulfate

**Fig. 3:**
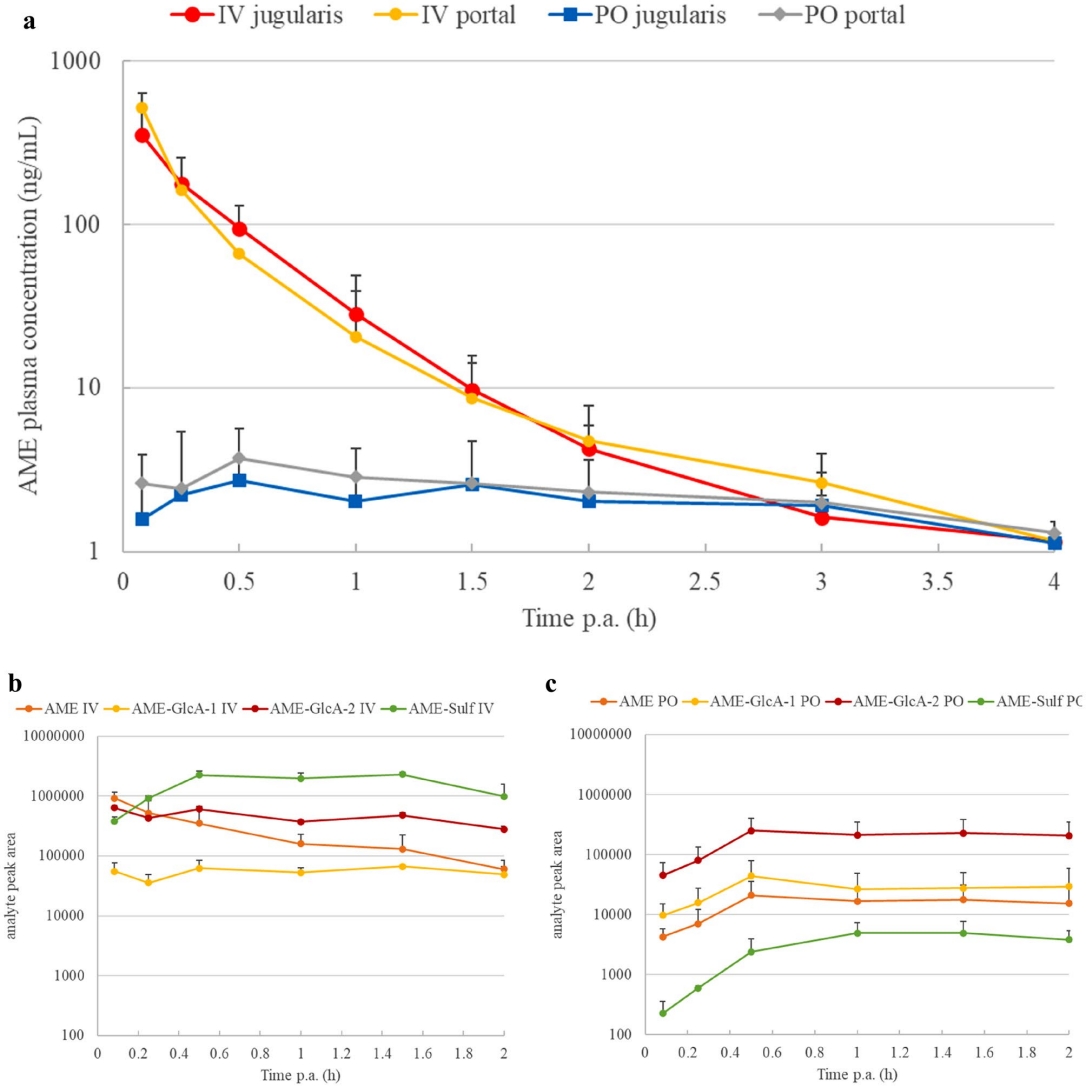
**a** Plasma concentration–time profiles (log-scaled) of alternariol monomethyl ether (AME) in piglets (*n* = 6) after a single intravenous (IV) and oral (PO) bolus dose administration of 2 mg/kg b.w. Blood was collected from both the *vena jugularis* (jugularis) and *vena portae* (portal). Values are presented as mean + standard deviation. **b** and **c** show the major phase II metabolite profile in jugular plasma after IV and PO dosing, respectively, and expressed as absolute chromatographic peak area (mean + standard deviation). AME-GlcA: AME-glucuronide; AME-Sulf: AME-sulfate

The plasma concentration (log-scaled)–time profiles of AOH and AME are depicted in Fig. 2a and 3a, respectively. For both IV and PO dosing, the profiles obtained in jugular and portal plasma are shown. Interestingly, for each mycotoxin and within the same route of administration, the jugular and portal plasma profiles are similar. Since after IV administration only the liver is involved as biotransformation site, this indicates the liver is the major site for presystemic biotransformation, as suggested by Louro et al. (2024). Table 2 shows the major toxicokinetic parameter estimates of AOH and AME, based on the jugular plasma profiles. Mean AUC_0−*∞*_ was 125.6 and 19.1 ng*h/mL for AOH, and 132.8 and 9.43 ng*h/mL for AME after IV and PO administration, respectively. Consequently, both AOH and AME have a low absolute oral bioavailability of 15 and 9%, respectively. This indicates a low absorption and/or extensive first-pass biotransformation in the gut and/or liver. These low bioavailabilities corroborate the data of radiolabeled AOH in mice (Schuchardt et al. 2014) and radiolabeled AME in rats (Pollock et al. 1982), both reporting an oral bioavailability of < 10%. In addition, a rather rapid oral absorption takes place, since mean C_max_ values were reached at 2.2 h p.a. for AOH and 1.1 h p.a. for AME (Table 2).

**Table 2:** Major toxicokinetic parameters of alternariol (AOH) and alternariol monomethyl ether (AME) after single intravenous (IV) and oral (PO) administration (2 mg/kg b.w.) to piglets (*n* = 6, except *n* = 3 for f_e_)

	AOH		AME	
Toxicokinetic parameter	IV	PO	IV	PO
AUC_0-∞_ (ng*h/mL)	125.6 ± 8.3	19.1 ± 17.7	132.8 ± 44.3	9.43 ± 7.34
F (%)	–	15 ± 13	–	9 ± 11
C_0_ (ng/mL)	903.1 ± 515.7	–	487.8 ± 266.9	–
C_max_ (ng/mL)	–	4.02 ± 4.06	–	2.83 ± 2.85
t_max_ (h)	–	2.2 ± 2.5	–	1.1 ± 1.2
t_1/2el_ (h)	0.16 ± 0.06	–	0.21 ± 0.08	–
k_e_ (h^−1^)	5.07 ± 2.19	–	3.61 ± 1.31	–
CL (L/(h*kg))	12.9 ± 4.83	–	16.8 ± 7.05	–
V_d_ (L/kg)	4.97 ± 3.30	–	5.15 ± 2.71	–
f_e_ (%)	3.79 ± 1.44	–	9.74 ± 6.24	–

Values are presented as mean ± standard deviation

*AUC*_*0-∞*_ area under the plasma concentration–time curve from time 0 to infinity, *F* absolute oral bioavailability, *C*_*0*_ plasma concentration at 0 h after IV dosing, *C*_*max*_ maximal plasma concentration after PO dosing, *T*_*max*_ time at which C_max_ occurs, *t*_*1/2el*_ plasma elimination half-life; *ke* elimination rate constant, *CL* total body clearance, *V*_*d*_ volume of distribution at steady state, *f*_*e*_ urinary fraction excreted unchanged

**Fig. 4:**
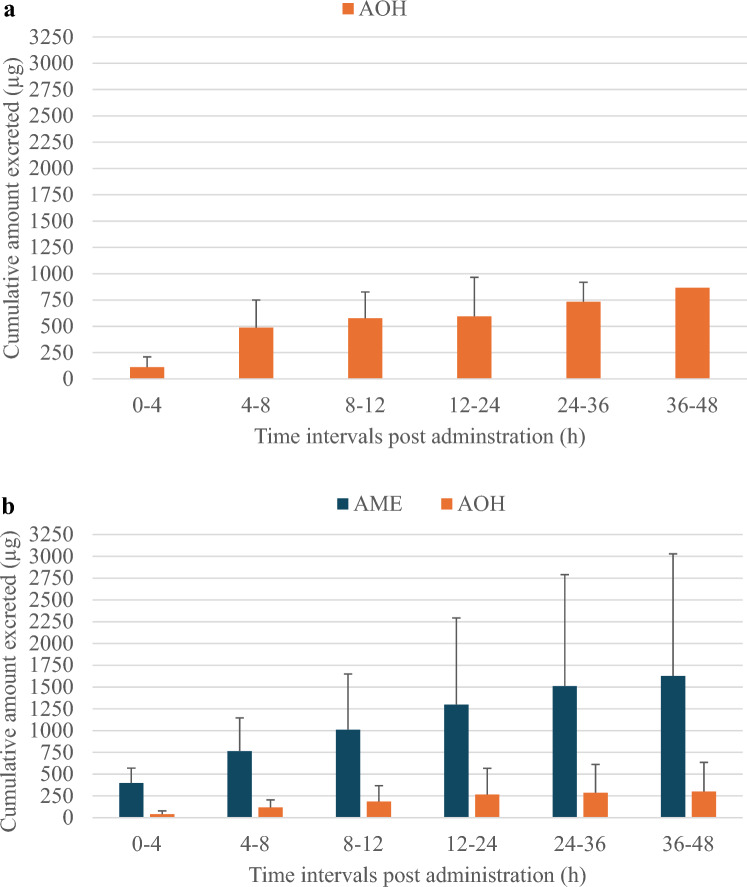
Cumulative urinary excretion profiles of alternariol (AOH) and alternariol monomethyl ether (AME) in piglets after a single intravenous (IV) bolus dose administration of 2 mg/kg b.w. of AOH (Fig. 4a, *n* = 3) and AME (Fig. 4b, *n* = 3). Values are presented as mean + standard deviation

The quantitative cumulative urinary excretion profiles of AOH and AME are shown in Fig. 4. The higher SD values for AME compared to AOH reflect more inter-pig variation in the urinary excretion of AME *vs* AOH, although only 3 piglets were included which might at least partly explain this variability. Table 2 shows a low urinary fraction excreted unchanged (f_e_) after IV dosing for both mycotoxins, with a mean value of 3.8 and 9.7% for AOH and AME, respectively. Although literature data using IV dosing in pigs is lacking, oral radiolabeled AOH and AME were recovered with 9.3% in the urine of NMRI mice (Schuchardt et al. 2014) and 7% in the urine of Sprague Dawley rats (Pollock et al. 1982), respectively. The low fe results after IV dosing in our study suggest efficient biotransformation in pigs, and is demonstrated further by the high CL of 12.9 L/(h*kg) for AOH and 16.8 L/(h*kg) for AME, respectively (Table 2). Moreover, Fig. 4 shows the presence of AOH in urine after IV AME administration, suggesting *O*-demethylation of AME, as reported by Pollock et al. (1982) in vitro using rat liver S9 fraction with reduced nicotinamide adenine dinucleotide phosphate (NADPH) as coenzyme. Based on the cumulative urinary excretion data of AOH obtained after IV AME administration, the mean relative amount of AOH formed was 15.8%. This confirms the in vitro findings using hepatic microsomes from rats, humans and pigs, reporting the amount of AOH formed was about 20% in all three species after incubation of 50 µM AME for 40 min (Pfeiffer et al. 2007).

The body distribution of a compound is described by its V_d_, and is influenced by its physicochemical characteristics (such as pKa, log P, molecular weight), plasma and tissue protein binding, and body composition such as the water/fat ratio (Gasthuys et al. 2016). High mean V_d_ values of 4.97 L/kg and 5.15 L/kg are obtained for AOH and AME, respectively (Table 2). These indicate substantial systemic exposure, although the high CL values are also responsible for a rapid plasma elimination of both mycotoxins, with a mean k_e_ of 5.07/h for AOH and 3.61/h for AME, respectively, and a short mean t_1/2el_ of only 0.16 h for AOH and 0.21 h for AME, respectively.

These quantitative AOH and AME toxicokinetic data are in marked contrast to the toxicokinetics of other *Alternaria* mycotoxins, such as TeA, obtained by our group in pigs. Using a similar IV and PO crossover design in piglets, Fraeyman et al. (2015) reported a complete absolute oral bioavailability and a low CL of 0.45 L/(h*kg) for TeA. Both findings point towards a limited biotransformation of TeA. Interestingly, some human in vivo data of TeA are available to compare: 2 volunteers orally ingested a dose of TeA of 30 µg and 90% of the dose was excreted unchanged in urine (Asam et al. 2013), confirming the high bioavailability of TeA our group reported in pigs. The V_d_ of TeA in pigs was low, with a mean value of 0.33 L/kg. When comparing V_d_ data for other mycotoxins in pigs using a similar IV study design, a value of 0.61 L/kg was reported by our group for DON (Broekaert et al. 2017) and 7.3 L/kg for ZEN (Catteuw et al. 2019). This correlates well with log *P* values, with the more hydrophobic mycotoxins diffusing more easily and having a larger V_d_ (log *P* values for DON: −0.7; TeA: 1.2; AOH: 2.9; AME: 3.2, ZEN: 3.6, respectively (PubChem 2024)). Moreover, the CL values of these other mycotoxins can be compared based on our previous IV data in pigs, showing marked differences. Indeed, a CL of 0.32 L/(h*kg) was reported for DON (Broekaert et al 2017), in contrast to a much higher value of 6.7 L/(h*kg) for ZEN (Catteuw et al. 2019). Of relevance, the efficient biotransformation of ZEN to its more potent alpha-zearalenol metabolite in pigs is well known and is responsible for the bioactivation and oestrogenic properties of this mycotoxin in pigs (Tiemann and Dänicke 2007).

Eventually, an ADMET evaluation can be performed by combining these in vivo ADME properties with published in vitro toxicity data for the 3 emerging *Alternaria* mycotoxins AOH, AME and TeA. Using cytotoxicity as a toxicological endpoint, it can be concluded that AOH and AME having the highest in vitro cytotoxicity as previously reported by our group and also reviewed recently (den Hollander et al. 2022; Louro et al. 2024), show the lowest systemic exposure due to the limited oral absorption and/or extensive first-pass biotransformation. In contrast, TeA having lower in vitro cytotoxicity, shows a high systemic exposure due to complete oral absorption and limited biotransformation (Fraeyman et al. 2015). However, a limited oral absorption of AOH and AME may implicate a higher impact on the gut barrier and health, which may be a relevant subject for further study.

### Metabolite profiling in urine and plasma

**Fig. 5:**
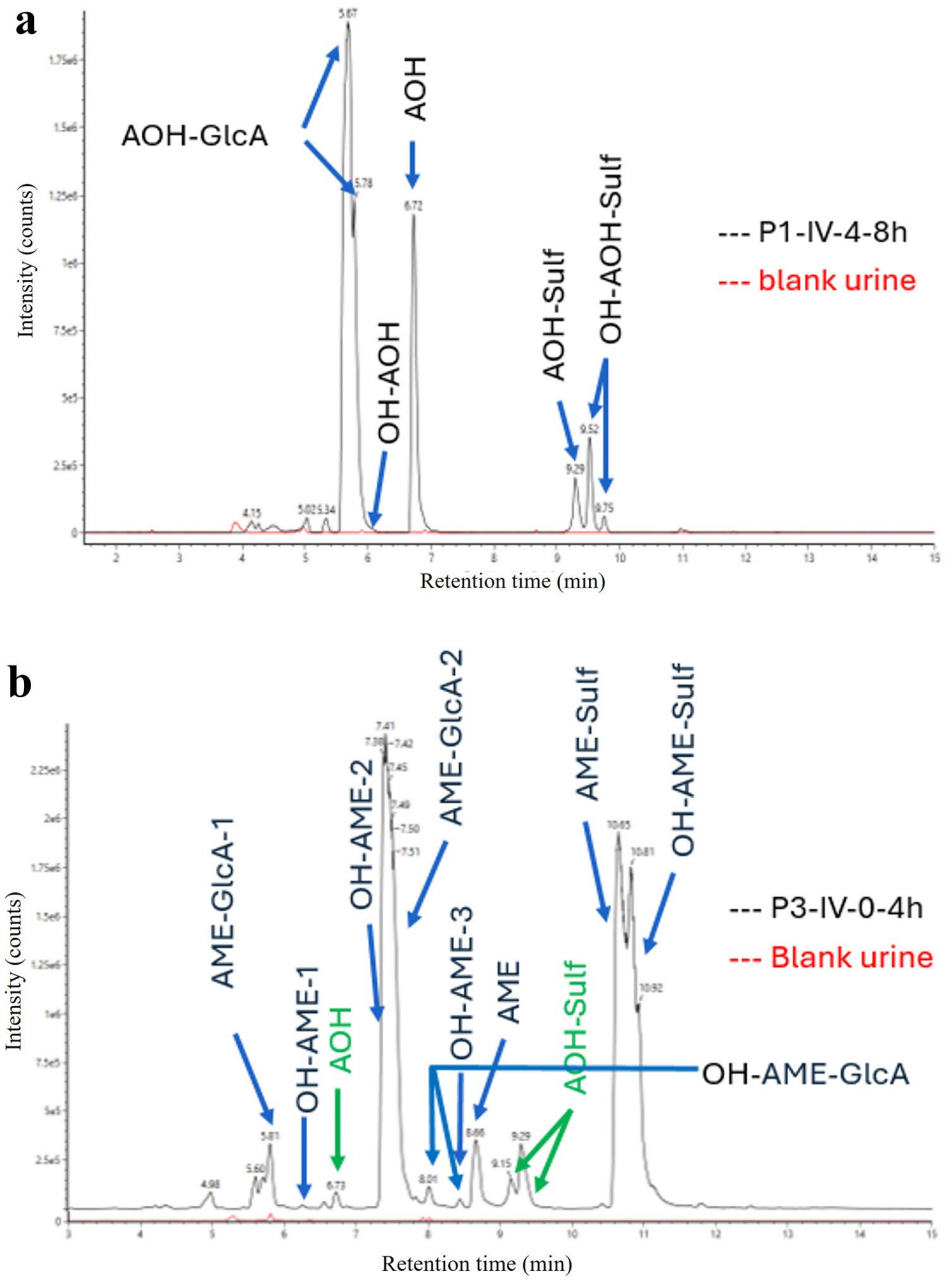
LC-HRMS extracted ion chromatograms of **a** a urine sample collected from pig n° 1 in the period 4–8 h after a single intravenous (IV) bolus dose administration of 2 mg/kg b.w. of alternariol (AOH) and a blank urine sample (red trace); and **b** a urine sample collected from pig n° 3 in the period 0–4 h after single intravenous (IV) bolus dose administration of 2 mg/kg b.w. of alternariol monomethyl ether (AME) and a blank urine sample (red trace)

**Fig. 6:**
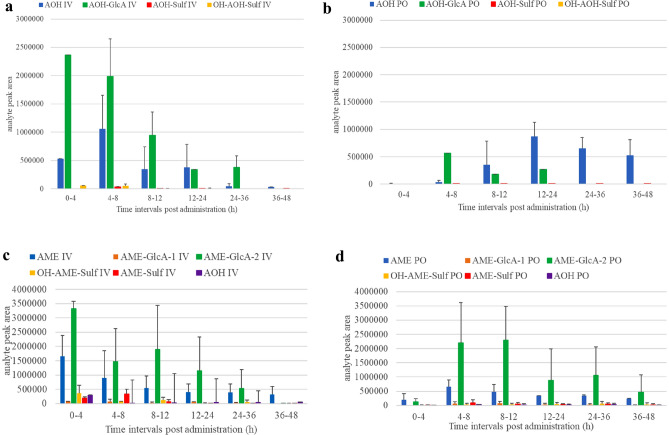
Metabolite profile in pig urine after a single intravenous (IV) and oral (PO) bolus dose administration of 2 mg/kg b.w. of alternariol (AOH) (Fig. 6a and b, respectively, *n* = 3) and alternariol monomethyl ether AME (Fig. 6c and d, respectively, *n* = 3). Values are presented as absolute chromatographic peak area (mean + standard deviation). AOH-GlcA: AOH-glucuronide; AOH-Sulf: AOH-sulfate; OH-AOH-Sulf: hydroxy-AOH-sulfate; AME-GlcA: AME-glucuronide; AME-Sulf: AME-sulfate; OH-AME-Sulf: hydroxy-AME-sulfate

Representative LC-HRMS extracted ion chromatograms of a urine sample from a pig after IV administration of AOH and AME, respectively, are shown in Fig. 5a and b together with a blank urine sample. Extensive biotransformation can be observed to mainly phase II metabolites, i.e. glucuronide conjugates (AOH-GlcA; AME-GlcA-1 and -2), and sulfate conjugates of parent AOH and AME as well as hydroxylated AOH and AME (AOH-Sulf and OH-AOH-Sulf; AME-Sulf and OH-AME-Sulf). In Fig. 6, these major metabolites are presented as urinary metabolite profiles for each urine collection interval after IV and PO dosing, together with AOH and AME, and expressed as mean chromatographic peak areas. For AOH, mainly one chromatographic peak of its glucuronide conjugate was detected, whereas for AME two distinct (i.e. baseline-separated) glucuronide conjugates were present. This is consistent with in vitro assays, as reviewed recently by Louro et al. (2024). Indeed, in the presence of uridine diphosphate glucuronic acid (UDPGA), in liver microsomes of sows as well as of female Sprague Dawley rats, and in intestinal microsomes of sows and humans, the AOH-3-*O*-GlcA conjugate was most prevalent, while in hepatic and intestinal microsomes of male Sprague Dawley rats, the AOH-9-*O*-GlcA predominated (Pfeiffer et al. 2009). Regarding AME, in vitro assays showed AME-3-*O*-GlcA as the major glucuronide in rats, pigs and humans, while AME-7-*O*-GlcA was also present but to a lesser extent (Pfeiffer et al. 2009). When AOH was incubated with rat liver cytosol in the presence of 3’-phosphoadenosine-5’-phosphosulfate (PAPS), AOH-3-*O*-Sulf, AOH-7-*O*-Sulf and AOH-9-*O*-Sulf conjugates were detected, while AME-3-*O*-Sulf and AME-7-*O-*Sulf were detected (Burkhardt et al. 2009).

The LC-HRMS extracted ion chromatograms of urine samples also show some mono-hydroxylated phase I metabolites (OH-AOH; OH-AME-1, -2 and -3). Phase I hydroxylation may take place at all 4 possible positions on the aromatic ring structures of both AOH and AME (at C2, C4, C8 and C10, Fig. 1b), as observed with hepatic rat, human and porcine microsomes (Pfeiffer et al. 2007), as well as with human recombinant cytochrome P450 isoforms (Pfeiffer et al. 2008) and rat liver slices (Burkhardt et al. 2011). Still, recently Borsos et al. (2024) showed some interspecies differences in the pattern and rate of phase I biotransformation. It should be mentioned that the mono-hydroxylated metabolites are catechols or hydroquinones, and may thus be of toxicological relevance. Indeed, catechols are suspected to form reactive intermediates such as quinones and semiquinones, resulting in subsequent DNA adducts and the production of reactive oxygen species (EFSA Panel on Contaminants in the Food Chain 2011). Other biotransformation reactions included in our analysis (Table S3) were not detected.

Concerning plasma metabolite profiling, the chromatographic peak area (log-scaled)–time profiles of major metabolites as detected in urine are shown in Fig. 2b,c and 3b,c for AOH and AME, respectively. More specifically, for both mycotoxins the profile in jugular plasma after IV and PO dosing is presented. The profiles in portal plasma were very similar (data not shown). The curves presented for the peak area of the parent compounds AOH and AME confirm the data of our quantitative UPLC-MS/MS analysis since the absolute oral bioavailability is very low. Mainly phase II conjugates were detected and were already present in porcine plasma at the first sampling point. Moreover, these profiles show some marked differences according to the route of administration. After IV dosing, both glucuronide and sulfate conjugates were detected for both mycotoxins, whereas after PO dosing the glucuronide conjugates were predominant. Biotransformation during first pass is less efficient in sulfate conjugation. Indeed, the mean ratios of the peak areas of parent/metabolite showed a noticeable difference between the two application routes, as calculated for the samples collected during the first 2 h p.a. The mean AOH/metabolite ratios were 0.03 and 0.08 for AOH-Sulf and AOH-GlcA, respectively after IV application, and 0.81 and 0.02, respectively after PO application. For AME these ratios were 0.56 and 0.69 for AME-Sulf and AME-GlcA-2, respectively after IV application, compared to 8.39 and 0.08, respectively after PO dosing. Thus, it appeared that mainly glucuronide conjugation is responsible for the presystemic metabolism when AOH and AME are absorbed from the gut.

In summary, our results show that in vivo in pigs, AOH as well as AME and their hydroxylated metabolites are predominantly present as phase II conjugates with glucuronic acid and/or sulfate. Some of the biotransformation pathways seen in our study were also reported in vivo in other species. In mice, the presence of all 4 hydroxylated phase I metabolites was noted (Schuchardt et al. 2014), as well as AOH-3-*O*-Sulf in rats (Puntscher et al. 2019), although the latter study did not investigate glucuronides. In a recent study in humans (Visintin et al. 2024), 3 volunteers ingested a single oral bolus of AOH and AME at the TTC dose. Mainly after enzymatic hydrolysis, AOH and AME could be quantified in some urine samples, pointing towards the presence of phase II conjugates in humans and their potential role as candidate biomarkers of exposure in biofluids and biomonitoring studies (Visintin et al. 2024).

## Conclusion

In pigs, the absolute oral bioavailability of both AOH and AME was shown to be low, due to limited absorption and/or extensive first-pass biotransformation, mainly in the liver. This biotransformation consists of moderate phase I (mainly oxidative metabolites) and extensive phase II (glucuronidation and sulfation) reactions. In accordance, quantitative toxicokinetic modeling revealed that both AOH and AME have a high CL and V_d_, and a short t_1/2el_. These toxicokinetic results may contribute to the risk assessment of AOH and AME in pigs and humans, as pigs can serve as surrogate animal model. The metabolite profiling can inform the development of biomarkers for exposure in pigs and future biomonitoring studies in humans.

## Supplementary Information

Below is the link to the electronic supplementary material.

Supplementary file1 (DOCX 29 KB)

## Data Availability

Data are located in controlled access data storage at Ghent University.

## Abbreviations

AA: Acetic acid
ACN: Acetonitrile
ADME: Absorption, distribution, metabolism, excretion
AME: Alternariol monomethyl ether
AME-d4: Alternariol monomethyl ether-d4 [^2^H4]
AOH: Alternariol
AOH-d4: Alternariol-d4 [^2^H4]
Ar,∞: Cumulative amount excreted in urine
AUC: Area under the curve
b.w.: Body weight
C0: Plasma concentration at time 0
CL: Total body clearance
C_max_: Maximal plasma concentration
DMSO: : Dimethyl sulfoxide
DON: Deoxynivalenol
EC: European commission
EFSA: European food safety authority
EMA: European medicines agency
ESI: Electrospray ionization
EtAc: Ethyl acetate
EU: European union
FA: Formic acid
f_e_: Urinary fraction excreted unchanged
GlcA: Glucuronic acid or glucuronide
gof: Goodness-of-fit coefficient
HCl: Hydrochloric acid
ICH: International council for harmonisation of technical requirements for pharmaceuticals for human use
IS: Internal standard
IV: Intravenous
k_e_: Elimination rate constant
LC-HRMS: Liquid chromatography-high-resolution mass spectrometry
LOD: Limit of detection
LOQ: Limit of quantification
*m/z*: Mass-to-charge ratio
MeOH: Methanol
MRM: Multiple reaction monitoring
NADPH: Nicotinamide adenine dinucleotide phosphate
NH_4_FA: Ammonium formate
p.a.: Post administration
PAPS: 3’-Phosphoadenosine-5’-phosphosulfate
PBPK: Physiologically based pharmacokinetic modeling
PO: Per os
R: Correlation coefficient
RSD: Relative standard deviation
SD: Standard deviation
S/N: Signal-to-noise ratio
Sulf: Sulfate
t_1/2el_: Elimination half-life
TeA: Tenuazonic acid
t_max_: Time to maximal plasma concentration
TOF: Time-of-flight
T_R_: Retention time
TTC: Threshold of toxicological concern
UDPGA: Uridine diphosphate glucuronic acid
UPLC-MS/MS: Ultra-performance liquid chromatography − tandem mass spectrometry
V_d_: Volume of distribution
VICH: International cooperation on harmonisation of technical requirements for registration of veterinary medicinal products
WS: Working solution
ZEN: Zearalenone
