# Supplementary material for: Absolute oral bioavailability, quantitative toxicokinetics and metabolite profiling of alternariol and alternariol monomethyl ether in pigs
Source: Arch Toxicol. 2025 Apr 26;99(7):2801–17. doi: 10.1007/s00204-025-04050-y (PMC12198339; doi:10.1007/s00204-025-04050-y)
Supplement: Supplementary file 1 — Supplementary file1 (DOCX 29 KB) [file 204_2025_4050_MOESM1_ESM.docx]

**Absolute oral bioavailability, quantitative toxicokinetics and metabolite profiling of alternariol and alternariol monomethyl ether in pigs**

Danica den Hollander^1a^, Siegrid De Baere^1a^, Celestien Holvoet^1,2^, Mathias Devreese^1^, Gunther Antonissen^1,3^, Ann Martens^4^, Kristel Demeyere^2^, Kris Audenaert^5^, Evelyne Meyer^2^ and Siska Croubels^1^*

^1^ Laboratory of Pharmacology and Toxicology, Department of Pathobiology, Pharmacology and Zoological Medicine, Faculty of Veterinary Medicine, Ghent University, Salisburylaan 133, 9820 Merelbeke, Belgium

^2^ Laboratory of Biochemistry, Department of Veterinary and Biosciences, Faculty of Veterinary Medicine, Ghent University, Salisburylaan 133, 9820 Merelbeke, Belgium

^3^ Chair Poultry Health Sciences, Department of Pathobiology, Pharmacology and Zoological Medicine, Faculty of Veterinary Medicine, Ghent University, Salisburylaan 133, 9820 Merelbeke, Belgium

^4^ Department of Large Animal Surgery, Anaesthesia and Orthopaedics, Faculty of Veterinary Medicine, Ghent University, Salisburylaan 133, 9820 Merelbeke, Belgium

^5^ Laboratory of Applied Mycology and Phenomics, Department of Plants and Crops, Faculty of Bioscience Engineering, Ghent University, Valentin Vaerwyckweg 1, 9000 Gent, Belgium

^a^shared first author; *corresponding author Tel: +32 9 264 73 45; Fax: +32 9 264 74 97; Email: [siska.croubels@UGent.be](mailto:siska.croubels@UGent.be)

**Supplementary Tables**

**Table S1** Results of the evaluation of the linearity (calibration range, correlation coefficient (r), goodness-of-fit coefficient (gof)), limit of detection (LOD) and limit of quantification (LOQ) for alternariol (AOH), alternariol monomethyl ether (AME) in pig plasma and urine. Experiments were performed over 3 days and are expressed as mean ± standard deviation

| **Analyte and matrix** | **Calibration range (ng/mL)** | **r^a^** | **gof^a^ (%)** | **LOD^c^ (ng/mL)** | **LOQ (ng/mL)** |
| --- | --- | --- | --- | --- | --- |
| AOH - plasma | 1 to 20 | 0.9970 ± 0.0014 | 5.8 ± 1.5 | 0.02 | 1.0 |
| AME - plasma | 1 to 20 | 0.9982 ± 0.0015 | 4.3 ± 2.1 | 0.01 | 1.0 |
| AOH - urine | 50 to 2,000 | 0.9965 ± 0.0017 | 6.8 ± 1.9 | 12.7 | 50 |
| AME - urine | 10 to 2,000 | 0.9998 ± 0.0013 | 4.4 ± 2.7 | 0.60 | 10 |

^a^acceptance criteria for linearity: r ≥ 0.99, gof ≤ 10 %; ^c^LOD : calculated concentration that corresponds to a signal to noise ratio (S/N) = 3

**Table S2** Results of the within-run and between-run accuracy and precision evaluation for the analysis of alternariol (AOH) and alternariol monomethyl ether (AME) in pig plasma and urine. Experiments for the between-run accuracy and precision were performed over 3 days. Results are expressed as mean ± standard deviation

| **Analyte and matrix** | **Spiked concentration**  **(ng/mL)** | **Mean found**  **concentration ± SD**  **(ng/mL)** | **Precision, RSD**  **(%)** | **Accuracy**  **(%)** |
| --- | --- | --- | --- | --- |
| AOH - plasma | 1.0^a^ (n = 6) | 1.02 ± 0.03 | 3.2 | 1.5 |
|  | 1.0^b^ (n = 16) | 1.04 ± 0.06 | 6.1 | 3.5 |
|  | 10.0^a^ (n = 6) | 10.3 ± 0.2 | 1.5 | 3.1 |
|  | 10.0^b^ (n = 16) | 11.1 ± 0.7 | 6.7 | 11.0 |
| AME - plasma | 1.0^a^ (n = 6) | 1.01 ± 0.02 | 1.5 | 1.2 |
|  | 1.0^b^ (n = 16) | 1.03 ± 0.04 | 4.0 | 3.2 |
|  | 10.0^a^ (n = 6) | 9.9 ± 0.1 | 0.5 | -1.4 |
|  | 10.0^b^ (n = 16) | 10.8 ± 0.8 | 7.4 | 8.0 |
| AOH - urine | 50.0^a^ (n = 6) | 48.0 ± 10.4 | 21.6 | -3.9 |
|  | 50.0^b^ (n = 16) | 49.5 ± 9.5 | 19.2 | -1.1 |
|  | 100.0^a^ (n = 6) | 105.3 ± 7.0 | 6.6 | 5.3 |
|  | 100.0^b^ (n = 18) | 104.1 ± 10.5 | 10.1 | 4.1 |
|  | 1,000.0^a^ (n = 6) | 1,038.6 ± 64.0 | 6.2 | 3.9 |
|  | 1,000.0^b^ (n = 18) | 1,091.8 ± 82.8 | 7.6 | 9.2 |
| AME- urine | 10.0^a^ (n = 6) | 10.5 ± 0.4 | 3.9 | 4.9 |
|  | 10.0^b^ (n = 17) | 10.9 ± 1.0 | 8.8 | 9.1 |
|  | 100.0^a^ (n = 6) | 94.3 ± 4.5 | 4.7 | -5.7 |
|  | 100.0^b^ (n = 18) | 96.7 ± 6.8 | 7.1 | -3.3 |
|  | 1,000.0^a^ (n = 6) | 965.5 ± 81.8 | 8.5 | -3.5 |
|  | 1,000.0^b^ (n = 18) | 1,007.3 ± 59.0 | 5.9 | 0.7 |

^a^within-run accuracy and precision (n ≥ 5); ^b^between-run accuracy and precision (at least 3 analytical runs over at least 2 days); SD: standard deviation; RSD: relative standard deviation; acceptance criteria for accuracy: LOQ: ± 20 %, other concentration levels : ± 15 %; acceptance criteria for precision (RSD_max_): LOQ: ± 20 %, other concentration levels : ± 15 % (ICH 2022)

**Table S3** Overview of the type of transformation, chemical formula, theoretical neutral mass, observed neutral mass, mass error values (in mDa and in ppm) and retention time for alternariol (AOH), alternariol monomethyl ether (AME) and metabolites that were observed in urine samples

| **Component** | **Type of transformation** | **Chemical formula** | **Theoretical neutral mass (Da)**^a^ | **Observed neutral mass (Da)** | **Mass error (mDa)** | **Mass error (ppm)** | **Retention time (min)** |
| --- | --- | --- | --- | --- | --- | --- | --- |
| AOH | Parent component | C14H10O5 | 258.0528 | 258.0532^b^ | 0.4 | 1.5 | 6.73 |
| AOH-GlcA | Phase II – glucuronide conjugation | C20H18O11 | 434.0849 | 434.0867^b^ | 1.8 | 4.1 | 5.67; 5.78 |
| AOH-Sulf | Phase II – sulfate conjugation | C14H10O8S | 338.0096 | 338.0092^b^ | -0.4 | -1.2 | 9.15; 9.29 |
| OH-AOH | Phase I – oxidation | C14H10O6 | 274.0477 | 274.0476^b^ | -0.1 | -0.4 | 6.04, 6.89 |
| OH-AOH-Sulf | Phase I – oxidation and Phase II – sulfate conjugation | C14H10O9S | 354.0046 | 354.0044^b^ | -0.1 | -0.3 | 9.52; 9.75 |
| AME | Parent component | C15H12O5 | 272.0685 | 272.0673^c^ | -1.2 | -4.3 | 8.66 |
| AME-GlcA | Phase II – glucuronide conjugation | C21H20O11 | 448.1006 | 448.1013^c^  448.1015^c^ | 0.7  0.9 | 1.6  2.1 | 5.78  7.58 |
| AME-Sulf | Phase II – sulfate conjugation | C15H12O8S | 352.0253 | 352.0211^c^ | -4.2 | -11.8 | 10.65; 10.81 |
| OH-AME | Phase I – oxidation | C15H12O6 | 288.0634 | 288.0632^c^  288.0628^c^ | -0.2  -0.6 | -0.7  -2.0 | 6.25  7.41 |
| OH-AME-GlcA | Phase I – oxidation and Phase II – glucuronide conjugation | C21H20O12 | 464.0955 | 464.0963^c^ | 0.8 | 1.8 | 8.01; 8.43 |
| OH-AME-Sulf | Phase I – oxidation and Phase II – sulfate conjugation | C15H12O9S | 368.0202 | 368.0154^c^ | -4.8 | -13.0 | 11.15 |

^a^Da = Dalton; ^b^based on the LC-HRMS extracted ion chromatograms (Fig. 5) of a urine sample collected from pig n° 1 in the period 4-8 h after a single intravenous (IV) bolus dose administration of 2 mg/kg b.w. of alternariol (AOH); and c) a urine sample collected from pig n° 3 in the period 0-4 h after single intravenous (IV) bolus dose administration of 2 mg/kg b.w. of alternariol monomethyl ether (AME)
